# Radiation Signature in Plasma Metabolome of Total-Body Irradiated Nonhuman Primates and Clinical Patients

**DOI:** 10.3390/ijms25179208

**Published:** 2024-08-25

**Authors:** Ales Tichy, Alana D. Carpenter, Yaoxiang Li, Gabriela Rydlova, Pavel Rehulka, Marketa Markova, Marcela Milanova, Vojtech Chmil, Amrita K. Cheema, Vijay K. Singh

**Affiliations:** 1 Department of Radiobiology, Military Faculty of Medicine, University of Defence, 662 10 Brno, Czech Republic; ales.tichy@unob.cz (A.T.);; 2 Biomedical Research Centre, University Hospital Hradec Králové, 500 05 Hradec Králové, Czech Republic; 3 Division of Radioprotectants, Department of Pharmacology and Molecular Therapeutics, F. Edward Hébert School of Medicine, Uniformed Services University of the Health Sciences, Bethesda, MD 20814, USA; 4 Armed Forces Radiobiology Research Institute, Uniformed Services University of the Health Sciences, Bethesda, MD 20814, USA; 5 Department of Oncology, Lombardi Comprehensive Cancer Center, Georgetown University Medical Center, Washington, DC 20057, USA; 6 Department of Molecular Biology and Pathology, Faculty of Military Health Sciences, University of Defence, 500 01 Hradec Králové, Czech Republic; 7 Department of Haematology and Blood Transfusion, University Hospital Na Bulovce, 128 00 Prague, Czech Republic; 8 Department of Biochemistry, Molecular and Cellular Biology, Georgetown University Medical Center, Washington, DC 2057, USA

**Keywords:** biomarkers, metabolomics, total-body irradiation, plasma, human, nonhuman primate

## Abstract

In the last decade, geopolitical instability across the globe has increased the risk of a large-scale radiological event, when radiation biomarkers would be needed for an effective triage of an irradiated population. Ionizing radiation elicits a complex response in the proteome, genome, and metabolome and hence can be leveraged as rapid and sensitive indicators of irradiation-induced damage. We analyzed the plasma of total-body irradiated (TBI) leukemia patients (n = 24) and nonhuman primates (NHPs; n = 10) before and 24 h after irradiation, and we performed a global metabolomic study aiming to provide plasma metabolites as candidate radiation biomarkers for biological dosimetry. Peripheral blood samples were collected according to the appropriate ethical approvals, and metabolites were extracted and analyzed by liquid chromatography mass spectrometry. We identified an array of metabolites significantly altered by irradiation, including bilirubin, cholesterol, and 18-hydroxycorticosterone, which were detected in leukemia patients and NHPs. Pathway analysis showed overlapping perturbations in steroidogenesis, porphyrin metabolism, and steroid hormone biosynthesis and metabolism. Additionally, we observed dysregulation in bile acid biosynthesis and tyrosine metabolism in the TBI patient cohort. This investigation is, to our best knowledge, among the first to provide valuable insights into a comparison between human and NHP irradiation models. The findings from this study could be leveraged for translational biological dosimetry.

## 1. Introduction

The increasing risk of radiological/nuclear exposures underlines the need for functional radiation biomarkers for afflicted populations. Although acute radiation syndrome (ARS) may present with radiation-specific symptoms such as nausea, vomiting, and erythema, there is a latent period before the physiological indications appear. In this case, radiation biomarkers are used to detect and estimate the levels of radiation exposure and can be used as a triage tool in mass casualty situations to separate the worried-well population from the exposed population [1].

Recent advances in both radiation biomarkers and metabolomics allow for a better understanding of the effects of radiation exposure on the human body. Radiation biomarkers are biological indicators of radiation exposure that can be used to measure the level of radiation exposure a person has experienced. One of the most rapidly evolving methods for finding biomarkers is metabolomics, which has been utilized to identify the molecular characteristics of numerous clinical conditions and therapeutic approaches [2,3]. Hence, combining radiation biology and metabolomics can provide valuable insight into the diagnosis and treatment of ARS.

The application of a high-resolution mass spectrometry-based metabolomic technology is anticipated to produce a panel of biomarkers rather than just a single molecule. Consequently, a multi-metabolite profile derived from the metabolomics platform can identify reliable biomarkers to predict radiation injury [3].

A number of studies have been reported with the goal of providing metabolomics-based molecular characterization of the qualitative and quantitative response to ionizing radiation (IR) exposure. Such studies on radiation metabolomics have frequently used murine and rat models [4,5,6,7,8]. More challenging, but more translational, are the studies performed on NHPs.

The objective of this report was to compare metabolomic profiles in blood plasma collected pre- and post-irradiation from leukemia patients and NHPs to investigate similarities and differences in radiation response. We performed global metabolomics on NHP plasma collected 7 days pre-irradiation and 24 h after 7.2 Gy total-body irradiation (TBI). In NHPs, such a dose would lead to approximately 70% mortality within 60 days (LD_70/60_) [9]. As the intended use of radiation on humans is prohibited in ethical research, our group has focused on leukemia patients since they undergo TBI as a part of their treatment, and they are possibly the best accessible comparison to NHPs for basic research. The results from the NHP plasma analysis were compared to plasma collected from leukemia patients receiving two doses of 2.0 Gy TBI within 24 h. Several common metabolites (cholesterol, bilirubin, and 18-hydroxycorticosterone) were identified, as well as a few significantly altered metabolic pathways. However, most metabolites were regulated differently in NHPs versus oncological patients, underlying the necessity to extrapolate any data from animal models to humans carefully.

## 2. Results

### 2.1. Metabolite Alterations in Plasma from TBI Leukemia Patients as Indicators of Radiation Exposure

**Figure 1:**
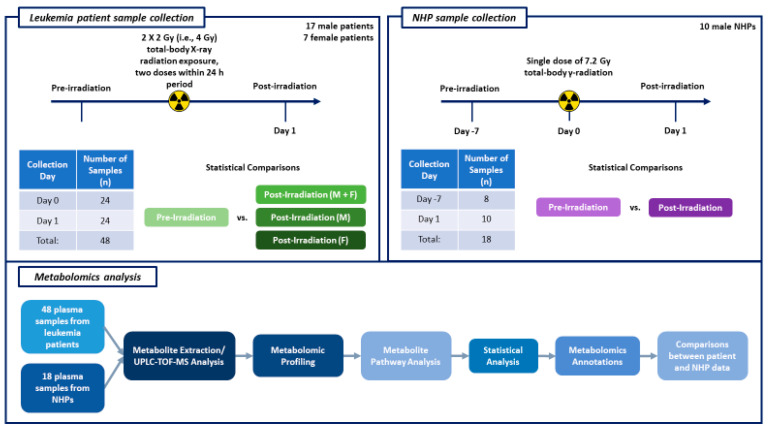
This figure illustrates the experimental design schema for the leukemia patient and NHP sample collection timelines, as well as the steps performed for metabolomics analysis.

Figure 1 details the workflow for metabolomics analysis.

**Figure 2:**
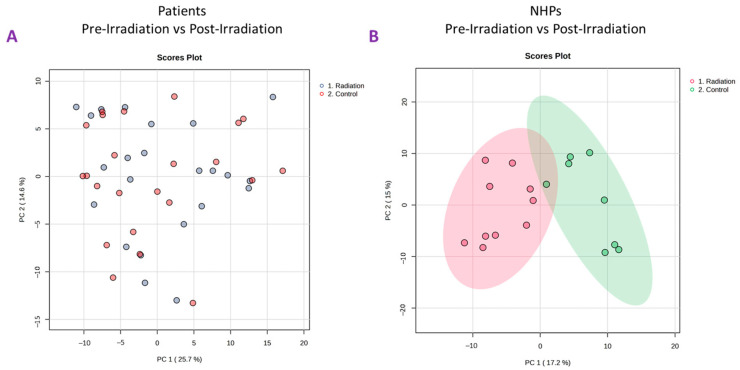
Principal Component Analysis (PCA) plots comparing pre-irradiation samples (control) to post-irradiation samples in total-body irradiated patients (panel (**A**)) and NHPs (panel (**B**)).

**Figure 3:**
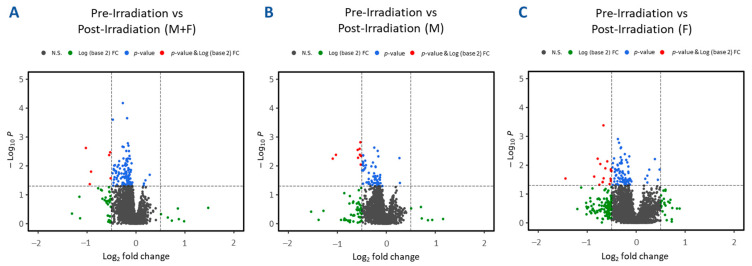
Volcano plots illustrating differential expression analysis in plasma samples collected from leukemia patients at various times pre-irradiation compared to post-irradiation (day 1). Panel (**A**) aggregates results from all human samples. Panels (**B**,**C**) further segregate human samples based on gender, showcasing male-only and female-only samples, respectively.

Plasma samples from 24 TBI patients were used for untargeted metabolomics using Waters Xevo G2-S Q-ToF. The plasma score plots (Figure 2A) demonstrate minimal grouping in the pre-irradiation and post-irradiation samples, suggesting a high degree of correlation between these two groups. A total of 3617 and 1188 features were identified in positive and negative electrospray ionization modes from human samples, respectively. The identity of significantly changed metabolites was verified by tandem MS. Volcano plots were constructed to assess the overall effects of radiation exposure (Figure 3A). Samples were further separated by gender, and volcano plots were constructed to view significant differences between males and females (Figure 3B and Figure 3C, respectively). Significant downregulation was noted in both males and females; however, female NHPs presented a higher degree of dysregulation post-irradiation compared to male NHPs. 

Plots demonstrate inter-group separation on the X-axis and intra-group variability on the Y-axis based on blood plasma profiles.

Next, we performed unpaired *t*-tests to delineate metabolites that significantly changed after exposure to IR (Supplementary Table S3). Significant differences (*p* ≤ 0.05) were observed in a few notable metabolites. Psychosine and 3,4-dihydroxyphenylacetate were significantly upregulated, while bilirubin, thyrotropin-releasing hormone, cholesterol, and 18-hydroxycorticosterone were significantly downregulated. Pathway analysis using MetaboAnalyst 6.0 revealed a limited number of significantly altered pathways in the patient samples. The identified compounds were involved in steroidogenesis, porphyrin metabolism, steroid biosynthesis, bile acid biosynthesis, and tyrosine metabolism (Table 1). In parallel, we also performed pathway enrichment analysis using the Mummichog analysis tool (Table 2), which underscored a minimal impact on metabolic processes as indicated by the current dataset. For this analysis, the altered metabolites in humans were linked to the C21-steroid hormone biosynthesis and metabolism, glycosphingolipid metabolism, and tyrosine metabolism pathways (*p* ≤ 0.05), suggesting that irradiation induced metabolic pathways related to inflammatory responses.

### 2.2. Metabolite Alterations in NHP Plasma as Indicators of Radiation Exposure

**Figure 4:**
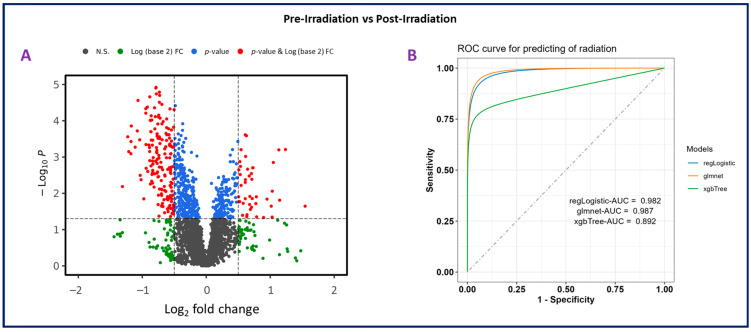
Panel (**A**). Volcano plot illustrating differential expression analysis in plasma samples collected from nonhuman primates (NHPs) pre-irradiation (day-7) compared to post-irradiation (day 1). The plot shows the log22 fold change versus −log10 *p*-value for each metabolite, highlighting significant changes. Panel (**B**). Receiver operating characteristic (ROC) curve for predicting radiation effects using an 8-metabolite panel. This analysis evaluates the ability to distinguish between the presence and absence of radiation exposure. Three models were used for prediction: regLogistic (AUC = 0.982), glmnet (AUC = 0.987), and xgbTree (AUC = 0.892).

Plasma samples from eight NHPs were subjected to untargeted metabolomics using high-resolution mass spectrometry (Xevo G2-S Q-ToF, Waters Corporation, Milford, MA, USA). The PCA plots (Figure 2B) revealed a significant difference between pre-irradiation and post-irradiation samples, as noted by the marked separation between groups (Figure 4A). A prediction model of radiation effects using an 8-metabolite panel including C00149, (S)-Malate; C00500, Biliverdin; C00219, (5Z,8Z,11Z,14Z)-Icosatetraenoic acid; C04598, 2-Acetyl-1-alkyl-sn-glycero-3-phosphocholine; C16596, 5-Phenyl-1,3-oxazinane-2,4-dione; C04230, 1-Acyl-sn-glycero-3-phosphocholine; C04317, 1-Organyl-2-lyso-sn-glycero-3-phosphocholine; and C00165, Diacylglycerol was constructed from NHPs and is shown in Figure 4B. The receiver operating characteristic (ROC) curve illustrates the ability to differentiate between the presence and absence of radiation exposure using three models: regLogistic, glmnet, and xgbTree. The glmnet model achieved the highest area under the curve (AUC, 0.987). This analysis supports the possibility of distinguishing radiation-exposed from non-exposed samples using this metabolite panel. A total of 3432 and 2866 features were observed in positive and negative electrospray ionization modes, respectively. The identity of considerably altered metabolites was validated by tandem MS. Next, unpaired *t*-tests were performed to identify metabolites that were significantly altered after radiation exposure. Notable metabolites were significantly downregulated (*p* ≤ 0.05), including S-(9-hydroxy-PGA2)-glutathione, estriol, coproporphyrinogen I, 12-oxo-c-LTB3, 2-acetyl-1-alkyl-sn-glycero-3-phosphocholine, 1-acyl-sn-glycero-3-phosphocholine, D-urobilinogen, aldosterone, 1-organyl-2-lyso-sn-glycero-3-phosphocholine, 18-hydroxycorticosterone, leukotriene D4, icosatetraenoic acid, and maleic acid. Conversely, 5-phenyl-1,3-oxazinane-2,4-dione; sterol; hydroxyandrost-5-en-17-one; 3(S)-hydroxy-10,13,16-all-cis-docosatrienoyl-CoA; bilirubin; biliverdin; urocortisol; prostaglandins E3 and G2; and diacylglycerol were significantly upregulated (Supplementary Table S4).

Pathway analysis using MetaboAnlayst revealed that the significantly dysregulated metabolites were related to steroid hormone biosynthesis, citrate cycle, porphyrin metabolism, glyoxylate and dicarboxylate metabolism, pyruvate metabolism, and arachidonic acid metabolism (Table 1). Some overlap was noted when using the Mummichog tool, which revealed that these significantly altered compounds were involved in C21-steroid hormone biosynthesis and metabolism, glycerophospholipid metabolism, tyrosine metabolism, prostaglandin formation from arachidonate, ascorbate and aldarate metabolism, porphyrin metabolism, TCA cycle, leukotriene metabolism, glycolysis and gluconeogenesis, omega-6 fatty acid metabolism, glycosylphosphatidylinositol-anchor biosynthesis, drug metabolism—cytochrome P450, and putative anti-inflammatory metabolites formation from eicosapentaenoic acid (Table 2).

## 3. Discussion

The current geopolitical situation concerning chemical, biological, radiological, and nuclear (CBRN) threads underscores the need for developing effective radiation countermeasures and reliable approaches for the diagnosis and treatment of ARS. Hence, the role of radiation biomarkers is indisputable, as the assessment of the appropriate parameters can reveal a person’s physiological status even before the symptoms manifest.

The gold standard in biodosimetry techniques based on cytogenetics (dicentric chromosome assay) is laborious and time-consuming. In addition, to use the metaphase spread, a calibration curve is required. The ring formation, an unstable chromosomal aberration, and a micronucleus assay, another well-known cytogenetic assay, are considered suitable cytogenetic assays for a mass-casualty scenario [10]. Furthermore, the premature chromosomal condensation assay identifying interphase cells has been demonstrated to discriminate between total- and partial-body exposures. Other conventional approaches (such as lymphocyte kinetics, nausea, etc.) lack specificity. Therefore, multiple radiation research groups have been engaged in the development of minimally invasive approaches. By studying metabolites as radiation biomarkers, one can understand how radiation affects the body on a molecular level and facilitate decisions about radiation safety by identifying individuals who may be at risk of developing long-term health effects due to radiation exposure.

We performed this global metabolomic study to identify potential radiation exposure markers in the plasma of leukemia patients and NHPs after TBI. Both leukemia patients and NHPs experienced metabolomic dysregulation post-irradiation; however, due to the differences in radiation doses used, leukemia patients did not experience dysregulation in terms of significantly dysregulated metabolites to the degree NHPs did. The untargeted screening of samples taken pre- and post-irradiation revealed a number of compounds that were significantly altered in both groups, including bilirubin, cholesterol, and 18-hydroxycorticosterone. Elevation of bilirubin in serum can be indicative of liver injury or hemolysis. It has been shown that bilirubin amplifies radiation injury and results in increased infection and death in mice [11]. Additionally, chronic exposure to ^137^Cs leads to increases in bilirubin and other compounds, such as cholesterol, in mouse models [12]. Interestingly, in our study, bilirubin was upregulated in NHPs but downregulated in humans. This implies that while results from NHPs are typically well translatable to humans, we should always consider their limited nature as they come from an experimental model; albeit, this difference could also be reflective of inherent differences in the two model systems.

Another metabolite was cholesterol, a biosynthetic precursor, which is a crucial component for membranes and lipid metabolism in general. It was found to be increased in atomic bomb survivors [13]. In vitro studies on lung cancer cell lines using high doses (5 Gy of X-rays) revealed that decreasing cholesterol by statin use is radioprotective, implying that increased cholesterol levels are correlated with cytotoxicity [14]. Interestingly, our results showed a modest decrease in cholesterol.

Finally, we found changes in 18-hydroxycorticosterone. When present in high concentrations, it can result in a serious electrolyte imbalance. No association with IR was found in the literature so far, but French et al. reported a rapid increase in 17-hydroxycorticosterone in rhesus macaques after TBI [15]; however, we found 18-hydroxycorticosterone to be downregulated in both humans and NHPs by 0.59-fold and 0.52-fold, respectively.

In NHPs, we found several metabolites with a previously reported association with IR. Despite the fact that the metabolites identified in NHPs and humans appeared to be two distinct sets at first, over-representation analysis revealed that these groups shared at least two metabolic pathways. The most significantly represented processes in both datasets were steroidogenesis (and steroid hormone biosynthesis and metabolism) and porphyrin metabolism.

The group of metabolites involved in steroid hormone metabolism comprised sterol, hydroxyandrost-5-en-17-one, estriol, and 18-hydroxycorticosterone. The role of hydroxyandrost-5-en-17-one (also known as dehydroepiandrosterone) [16] and estriol [17] in the response to irradiation has been previously reported. The other metabolite class associated with porphyrin metabolism comprised bilirubin, biliverdin, D-urobilinogen, and coproporphyrinogen I. Coproporphyrinogen I (a porphyrin metabolite) was previously detected in the urine of dogs after irradiation; however, the significant changes were found only after the lethal doses. Thus, it was not recommended as a suitable radiation biomarker. In that study, the excretion of urinary coproporphyrin was noted in dogs exposed to X-rays [18].

In NHPs, we also identified a group of compounds linked to eicosanoids: prostaglandins E3 and G2, leukotrien D4, and eicosatetraenoic acid (a leukotrien precursor). Prostaglandin E2 is known to facilitate recovery after hematopoietic ARS through increased hematopoietic stem cell survival [19]. Moreover, leukotrienes belong to a family of potent inflammatory mediators that appear to contribute to IR-induced pathophysiological processes [20].

That said, the candidate metabolomic radiation biomarkers could participate in the above-mentioned metabolic pathways. In addition, we detected three more compounds that function separately: diacylglycerol, S-(9-hydroxy-PGA2)-glutathione, and 12-oxo-c-LTB3. Diacylglycerol is involved in the T/B-cell receptor pathway. Nakajiwa and Yukawa reported that IR-induced hydroxyl radical generation is followed by diacylglycerol production and protein kinase C activation in rat hepatocytes [21]. The effect of radiation on glutathione levels is well described [22]. Finally, 12-Oxo-c-LTB3 belongs to the class of organic compounds known as hydroxyeicosatrienoic acids, which were identified as a part of a lipidomic biosignature in irradiated mice [23].

Several current metabolomic studies aiming to identify potential radiation biomarkers were performed using murine and NHP models, either with IR alone or in combination with medical countermeasures under development [6,7,8,24,25,26,27,28,29]. Metabolomic screening in the plasma of lethally irradiated NHPs revealed pathway perturbations that play a role in the preterminal stages: steroid hormone biosynthesis and glycerophospholipid metabolism pathways [30]. Both pathways are implicated in the inflammatory response and corroborate our findings. The steroid hormone biosynthesis pathway is of particular interest and has been shown to be significantly impacted by radiation, as demonstrated in other recently performed studies. The C21-steroid hormone biosynthesis and metabolism was the only pathway that was commonly significantly dysregulated by IR in four tissues (jejunum, lung, kidney, and spleen) collected from NHPs exposed to either TBI or PBI [29]. In a separate study, the steroid hormone biosynthesis pathway was significantly dysregulated in the plasma of irradiated NHPs [30]. As radiation is known to induce dyslipidemia, the effects of radiation on this pathway must be further investigated. Additionally, steroidogenesis and porphyrin metabolism might also be of particular interest for further investigation, since they seem to be crucial in radiation injury and have been shown to be dysregulated by IR in other metabolomic studies [24].

## 4. Materials and Methods

### 4.1. Experimental Design

Figure 1 provides the details of the sample collection timeline for both human patient and NHP studies, as well as the workflow for metabolomic analysis. We have also provided the days of sample collection in relation to radiation exposure and the number of samples collected.

### 4.2. Patient Study

#### 4.2.1. Leukemia Patients

Twenty-four leukemic patients with indicated TBI without previous or concurrent radiotherapy or chemotherapy were included in this study (Supplementary Table S1). Blood samples were collected from 17 males and 7 females aged from 19 to 58 years (median 40.5 years) undergoing TBI (two doses of 2.0 Gy delivered within 24 h). The informed consent from each individual was obtained for approval by the Ethics Committee of the University Hospital Bulovka, Prague, Czech Republic (protocol approval number 10/02/2017).

#### 4.2.2. Irradiation of Patients

Patients were irradiated by a standard C-arm linear accelerator, Versa HD (Elekta Solutions AB, Stockholm, Sweden), using homogenous 6 MV X-ray beams with a dose rate of 3 Gy/min. The doses were delivered to the patients within 24 h using the sweeping beam technique. In this method, the patient lies on a special couch situated on the floor perpendicular to the target–gun axis at a source-skin distance of 200 cm, and the linear accelerator gantry is sweeping over the patient with jaws wide open. To reach the TBI dosimetric recommendations regarding dose homogeneity, patients were irradiated by two arcs; the first arc was in a supine position and the second arc was in a prone position. Moreover, to maintain the constant source-to-patient distance, a U-like curved couch was used. Delivered doses were verified using in vivo measurements at several points on the skin in both positions—prone and supine.

#### 4.2.3. Blood Collection and Plasma Separation

All of the samples were collected at two different intervals: once prior to irradiation and 24 h after the second dose of radiation. The samples were collected into vacuum tubes (BD P100, Becton Dickinson, Franklin Lakes, NJ, USA) during the early morning and processed within one hour after collection. The plasma was separated by centrifugation (2500× *g* for 20 min at 22 °C), from which ~3.5 mL was aliquoted and stored at −80 °C.

### 4.3. NHP Study

#### 4.3.1. Animals and Animal Care

A total of 10 male naïve rhesus NHP (*Macaca mulatta*), 37–70 months of age, weighing 3.9–6.7 kg, were procured from the NIH Animal Center (Poolesville, MD, USA) and quarantined for at least 35 days prior to starting the experiment. Animal quarantine, housing, care, exclusion criteria, health monitoring, and enrichment have been discussed in detail previously [31]. All NHPs were given unique identification numbers prior to the initiation of the study by permanent tattoo. All experimental procedures were approved by the Armed Forces Radiobiology Research Institute/Uniformed Services University of the Health Sciences Institutional Animal Care and Use Committee (#2015-12-010) and the Department of Defense Animal Care and Use Review Office. This NHP study was accomplished in accordance with the recommendations in the Guide for the Care and Use of Laboratory Animals [32].

#### 4.3.2. Irradiation of NHPs

Under ketamine sedation (a dose of 5 to 15 mg/kg administered intramuscularly (*im*)), animals received a dose of 7.2 Gy ^60^Co gamma-radiation at a dose rate of 0.6 Gy/min. To deliver the exact dose of radiation, the lateral separations of the animals were measured prior to irradiation day, and animals were paired based on the lateral separations. The radiation field in the area of the animal placement was uniform (within ±1.5%). Dose measurements and calibrations (EMXmicro spectrometer, Bruker Corp., Billerica, MA, USA) were based on an alanine/EPR (electron paramagnetic resonance) system [33,34]. The alanine/EPR is considered one of the most accurate methods for radiation dosimetry (used by national standards laboratories for critical measurements and calibrations. All irradiation procedures and dosimetry were discussed in detail earlier [35]. After irradiation, animals were positioned in the transportation cart, returned to their home cages, and observed for their recovery from sedation.

#### 4.3.3. Blood Collection from NHPs and Plasma Separation

Blood samples were collected either from the saphenous or cephalic vein. Samples were collected 7 days prior to and 24 h post-irradiation. The blood sample collected in an EDTA (ethylenediaminetetraacetic acid) tube was then centrifuged (10 min, 400× *g*), and plasma was then transferred to a cryogenic storage tube. The plasma samples were stored at −80 °C until processed for metabolomic analysis.

### 4.4. Metabolite Extraction and LC-MS Analysis

Prior to sample preparation for LC-MS analysis, the sequence of samples was randomized to avoid any bias. Detailed methods for metabolite extraction and LC-MS analysis are described in detail in our recent publications using NHP samples [29,30].

### 4.5. Data Processing and Statistical Analysis

The untargeted (global) data attained were converted to the NetCDF (network common data form) unified data format using the Databridge tool in MassLynx mass spectrometry software V4.1 (Waters Corporation, Milford, MA, USA). The implementation of the XCMS R package (Scripps Institute, La Jolla, CA, USA) was used for peak detection as described earlier [36] using the IPO (Isotopologue Parameter Optimization) R package Version 1.30.0 [37]. The m/z_rt features (mass to charge ratio and retention time) were normalized with the internal standards (4-nitrobenzoic acid and debrisoquine in the extraction liquid), followed by data preprocessing steps, including logarithmic conversion and Pareto scaling. To identify metabolites with significant differential expression, we conducted statistical analysis using an unpaired *t*-test, which was corrected for multiple comparisons, setting a significance level at *p* < 0.05. The analysis of statistical data was conducted using MetaboAnalyst [38] version 6.0, while the pathway analysis was performed by Mummichog pathway and network analysis version 2.0.4 for metabolomics [39]. This analysis integrated molecular data into a biological context, providing a detailed overview of the systemic impacts of the metabolites examined. By merging statistical precision with biological pathway analysis, our method offers a refined interpretation of the findings, accounting for sample variability and yielding a deeper understanding of the biological implications. Supplementary Table S2 provides a complete list of the metabolites that were screened for in this study.

## 5. Conclusions

The focus of this study was to delineate radiation biomarkers using a metabolomics approach, enabling new insights into how exposure to radiation manifests at the molecular level.

The majority of metabolites were involved in pathways that were reported in other radiation metabolomics studies (metabolism of steroid hormones and porphyrin metabolism, among others), which underlies their biological significance. Another contribution of our study is that it identifies metabolites that can be further investigated in additional radiation-related studies. Despite several benefits of the metabolomic approach applied to radiation biomarker research, there are still some challenges that need to be addressed. There is a lack of expertise in this field, and the cost of equipment and supplies can be limiting as well. Our study bears some limitations as well. Ideally, it would be beneficial to study different radiation qualities and exposure types, but this would require a much larger cohort with long-term biobanking of samples. Also, irradiation of the leukemic patients with a higher dose (perhaps around 5 Gy) inducing a similar level of acute radiation syndrome would be useful for translational purposes and comparison with NHP. Additionally, samples from later time points would be valuable to assess the long-term effects of radiation exposure; however, the therapeutic regimen of two TBI doses per day does not allow such experiments in these kinds of patients.

Our results underline the importance of the temporal aspects of individual biomarkers. Some biomarkers might be useful only for certain time periods after irradiation, which has to be taken into account when designing the sampling strategy. To our knowledge, this report is the first attempt to provide a comparison of NHP and humans for TBI. The study is a comprehensive analysis of the radiation signature in the plasma metabolome of TBI patients and NHPs and provides valuable insights into the identification of potential radiation biomarkers and common metabolic pathways altered by ionizing radiation. The findings contribute to the development of metabolomic approaches for assessing radiation exposure and predicting radiation toxicity, addressing the critical need for effective radiation countermeasures and radiation biomarkers for practical biological dosimetry.

## Figures and Tables

**Table 1:** Over-representation analysis of enriched metabolites identified (MetaboAnalyst).

**Human Pathways**	**Total**	**Expected**	**Hits**	***p*-Value**	**FDR**
Steroidogenesis	43	0.17	2	0.010	0.051
Porphyrin metabolism	40	0.16	1	0.151	0.755
Steroid biosynthesis	48	0.19	1	0.179	0.895
Bile acid biosynthesis	65	0.26	1	0.236	1.000
Tyrosine metabolism	70	0.28	1	0.252	1.000
**NHP Pathways**	**Total**	**Expected**	**Hits**	***p*-Value**	**FDR**
Steroid hormone biosynthesis	87	0.39	3	0.005	0.030
Porphyrin metabolism	31	0.14	2	0.007	0.045
Citrate cycle (TCA cycle)	20	0.09	1	0.086	0.517
Pyruvate metabolism	23	0.10	1	0.099	0.591
Glyoxylate and dicarboxylate metabolism	32	0.14	1	0.135	0.810
Arachidonic acid metabolism	45	0.20	1	0.185	1.000

FDR: false discovery rate.

**Table 2:** Over-representation analysis of enriched metabolites (Mummichog).

Human Pathways	Overlap Size	Pathway Size
C21-steroid hormone biosynthesis and metabolism	4	26
Glycosphingolipid metabolism	1	4
Tyrosine metabolism	1	6
NHP pathways		
C21-steroid hormone biosynthesis and metabolism	10	35
Glycerophospholipid metabolism	9	29
Prostaglandin formation from arachidonate	5	6
Ascorbate (vitamin C) and aldarate metabolism	4	6
Porphyrin metabolism	4	8
TCA cycle	3	4
Leukotriene metabolism	3	6
Glycolysis and gluconeogenesis	2	2
Omega-6 fatty acid metabolism	2	3
Glycosylphosphatidylinositol-anchor biosynthesis	2	3
Drug metabolism—cytochrome P450	2	3
Putative anti-inflammatory metabolites formation from EPA	2	3

## Data Availability

All relevant data of this study are presented in the manuscript and its Supplementary files.

## Supplementary Materials

The following supporting information can be downloaded at: https://www.mdpi.com/article/10.3390/ijms25179208/s1.
